# Behaviour change approaches for individuals with diabetes to improve foot self-management: a scoping review

**DOI:** 10.1186/s13047-020-00440-w

**Published:** 2021-01-06

**Authors:** Joanne Paton, Sally Abey, Phil Hendy, Jennifer Williams, Richard Collings, Lynne Callaghan

**Affiliations:** 1 School of Health Professions, Faculty of Health, University of Plymouth, Derriford Road, Plymouth, PL6 8BH UK; 2 Torbay & South Devon NHS Foundation Trust, Torbay Hospital, Lowes Bridge, Torquay, TR2 7AA UK; 3 Penninsula Medical School, Faculty of Health, University of Plymouth, John Bull Building Plymouth Science Park, Plymouth, PL6 8BT UK

**Keywords:** Diabetic foot, Self-management, Behaviour change wheel, Foot ulcer prevention

## Abstract

**Background:**

Diabetes related foot complications are increasing in complexity, frequency and cost. The application of self-management strategies can reduce the risk of individuals developing foot complications. The type, range and nature of the literature focusing on interventions that support patients with diabetic foot self-management is unknown. This scoping review aimed to i) identify self-management actions and risky behaviour avoidance strategies within interventions, ii) map the theoretical functions through which these behaviour change interventions have an effect, iii) display gaps in the research.

**Methodology:**

Arksey and Malley’s (2003) 5 stage framework was followed to conduct the scoping study. This methodological framework was selected because it was developed specifically for scoping reviews and therefore offered clear methodological distinction from systematic review methodology. .

Databases were searched from inception of the project until June 2020 supplemented by hand searching of reference lists. In total 988 papers were identified. These were independently screened by three reviewers, identifying 19 eligible papers. Data extraction and charting of data was independently conducted by three reviewers to identify study characteristics, self-management actions and risky behaviours. Data was charted against the COM-B (capability, opportunity, motivation, behaviour) model of behaviour to determine intervention function.

**Results:**

In total 25 different foot self-management actions and risk behaviours were classified into three themes; routine self-management, trauma avoidance and warning signs and actions. Inspect feet daily received the most attention. The majority of interventions focused on knowledge and skills, but overlooked taking action and decision making. Intervention mapping identified four primary intervention functions (education, persuasion, training and enablement) used to address deficits in capability, opportunity and motivation that positively improved foot self-management behaviour. No studies targeted first ulcer prevention, and most either did not measure or improve foot health outcomes.

**Conclusion:**

This review charted the evidence for interventions promoting diabetic foot self-management through a theoretical behaviour change perspective. A core set of behaviour change activities and intervention functions associated with positive changes in behaviour were identified. This information will provide researchers with a useful basis for developing self-management interventions.

## Key message

- We recommend a more targeted, systematic approach to increase our understanding of the effectiveness of individual or clustered related foot care activities.
- Our exploratory findings may inform future intervention development. Clinically meaningful improvements in diabetic foot health seem to be associated with two combinations of intervention functions: 1. diabetic foot care education and foot care skills training delivered in combination with enablement (providing foot care equipment, prompts like daily diaries) Or 2. Diabetic foot care education and foot care skills training delivered in combination with persuasion (information to illicit an emotional response around what is good or bad foot care behaviour).
- Further research underpinned by a theoretical behaviour change framework, will help determine which combination of intervention functions are effective and cost-effective in supporting foot self-management behaviour for preventing foot ulceration.

## Introduction

Diabetes is a common long-term condition within the United Kingdom (UK) [1]. It is estimated that by 2025 the prevalence of diabetes will have doubled, from 2.7 million (2017) to over five million people [2, 3]. Every year, the National Health Service (NHS) undertakes over 7000 diabetic related lower limb amputations [4]. Eighty percent of these are preceded by preventable foot ulceration [5]. Fifty percent of people with diabetes who suffer a foot ulceration will not live beyond 5 years [6]. Between 31 and 36% of the diabetic population are classified at moderate or high-risk of developing a foot ulcer [3, 7]. Medical resources are concentrated on those with pre-existing foot pathology. Long-term foot health outcomes for people with diabetes could be improved through diabetic self-management strategies targeted toward people with diabetes, at risk of foot ulceration.

Diabetes UK, and the James Lind Alliance Priority Setting Partnership, identified ‘self-management in people with type 2 diabetes’ as a research priority [8]. The umbrella term ‘diabetic foot self-management’ includes a range of behaviour related self-management actions and risk-taking behaviour modifications that individuals are advised to perform on a regular basis. Most of the research evidence is focused upon those with diabetes required to self-manage their blood sugar control [9]. As the burden of diabetic complications, including foot ulceration, begins to outweigh the threat of poor glycaemic control, the focus has begun to shift toward strategies of self-management for diabetic foot ulcer prevention [10].

Engaging people with type 2 diabetes to take an active role in their daily foot self-management is important to promote good diabetic foot health, protect against diabetic foot ulceration and obtain early treatment for new foot ulceration [5]. Foot self-management involves foot health maintenance such as cutting nails regularly or drying between the toes [11–16]. Risk prevention measures include activities such as never walking barefoot, wearing shoes that fit, and foot health monitoring tasks, for example, self-screening for foot problems and contacting an appropriate health professional in the event of an injury [11–14, 17–20]. Some elements of foot self-management may be important for ulcer prevention and the maintenance of foot health. Diabetic foot self-management programmes are available to educate, support and enable people at moderate or high risk of developing a foot ulcer to alter their behaviour and self-manage their feet, however, uptake is low [21, 22].

Unfortunately many people with type 2 diabetes find it difficult to alter their behaviour and adhere to self-management regimes, often with devastating consequences to foot health. Reasons for none adherence are complex, such as patients considering foot care a lower priority than medication adherence or blood glucose control [23]. There is a misconception amongst those at greatest risk, that an absence of symptoms signals that nothing can be wrong [24]. Individuals with diabetic neuropathy often present with a dangerous lack of protective pain perception [25]. It is often difficult for individuals to comprehend their daily exposure to risk of tissue damage, or the potential benefit of foot care self-management [26] For many, it is only after experiencing the devastating consequences of diabetic foot ulceration first hand, that they become worried enough about their future foot health to engage in self-management to protect their feet for the future [23]. However, at this stage ulcer reoccurrence is often unavoidable. Approximately 40% of patients re-ulcerate within 1 year of their first ulcer, whilst 60% suffer a second ulceration within 3 years [27].

This scoping review maps the range of foot self-management behaviours included within diabetic foot self-management interventions investigated within the effectiveness studies.

**Fig. 1:**
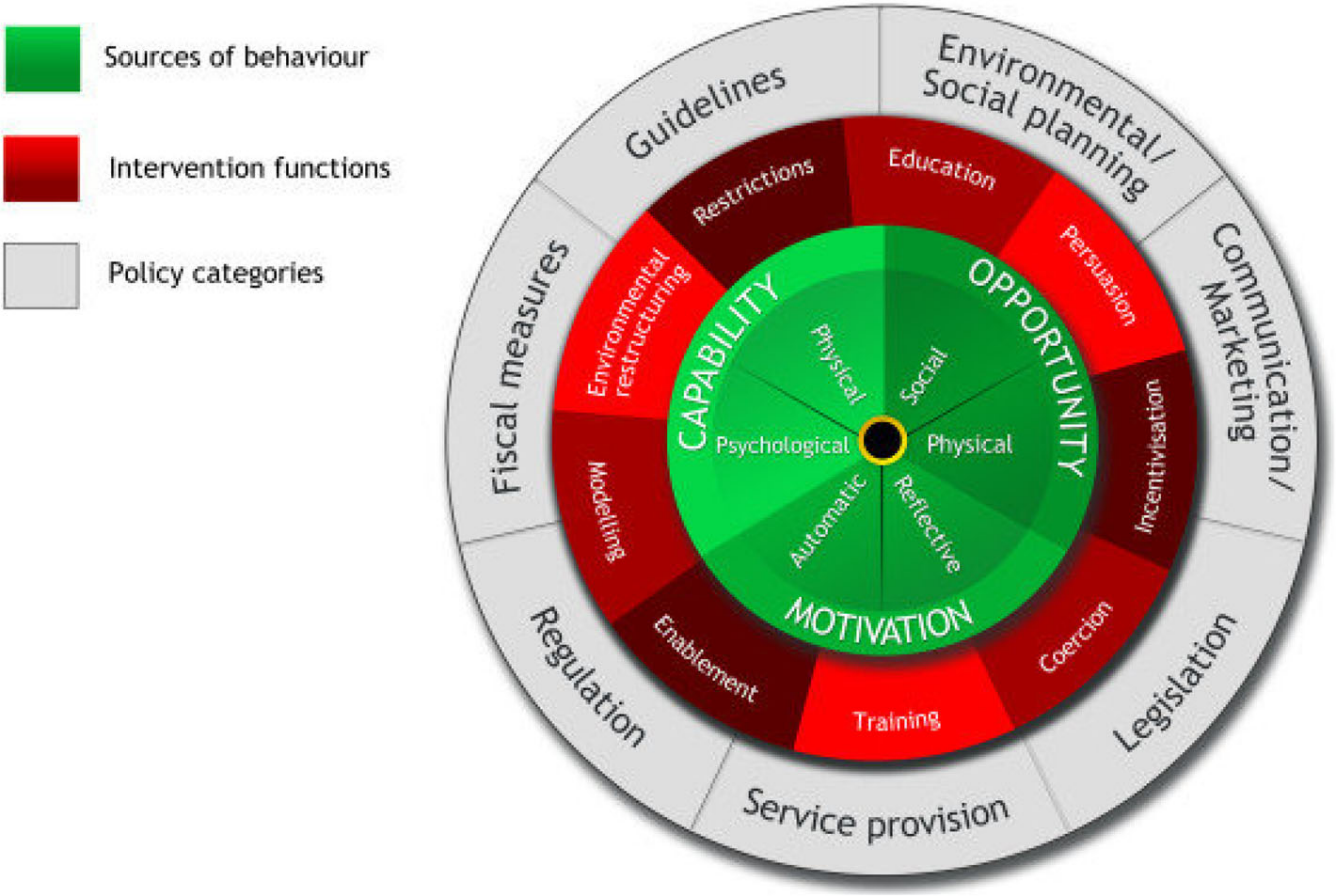
Behaviour Change Wheel COM-B Framework of Behaviour Change [29]

The second part of the review employs the COM-B framework [28] for behaviour change to characterise and compare the content of the diabetic foot self-management interventions (Fig. 1). The widely used COM-B framework was developed through the systematic review and selection of relevant aspects of other behaviour change tools. The COM-B framework was considered the most suitable framework for this review because of its dual purpose; 1. Providing explanation for why a recommended behaviour or intervention has not been implemented 2. Identifying domains that might be targeted as levers for change to inform intervention design. The psychology based COM-B model hypothesises that interaction between three components ‘capability’, ‘opportunity’ and ‘motivation’, best explains the variation in the success of the interventions devised to provoke change behaviour [10]. Through mapping the components of an intervention this review seeks to identify explanations for why a recommended behaviour is not implemented by an individual and identify domains that might inform the development of more effective interventions to support diabetic foot self-management by health professionals [10, 13].

### Aim

The objective of this scoping review is first to identify which of the recommended foot self-management actions or risky foot health behaviours have been the focus of evaluation studies designed to test interventions to prevent ulceration in people with diabetes, and second to map the COM-B model and intervention functions used within those interventions.

Research Questions:

1. What are the self-management actions or risky behaviour modifications targeted by researchers- evaluating behaviour change interventions for improving foot self-management for diabetic foot ulcer prevention and where are the gaps in research activity?
2. What are the specific intervention functions being implemented as part of the intervention and what conclusions have been drawn?
3. How are those interventions for improving foot self-management and reducing ulcer risk being implemented (context, target population, delivery format) and outcomes measured.

## Methods

### Protocol and registration

The protocol was drafted using the preferred reporting items for scoping reviews and meta- analysis protocols [30], and reviewed by the members of the research team. The protocol was registered retrospectively with the Open Science Framework on the 26th November 2019, (Registration number: osf.io/3ahsv).

The method employed to conduct the review followed the five stage framework described by Arksey and Malley [31] for conducting a scoping study and was reported in accordance with the PRISMA Extension for scoping reviews [32]. The inclusion and exclusion criteria for the scoping review was defined at the outset.

### Eligibility criteria

#### Inclusion criteria

The search was limited to articles written in English and adults over 18 years old, diagnosed with diabetes, without foot ulcer. Included were empirical studies evaluating the effectiveness of interventions directed towards the prevention of diabetic foot ulceration through the improvement of foot self-management or by the modification of risky foot health behaviour. Interventions reporting on outcome measures or analysis directed towards the modification of foot self-management behaviour were also included.

#### Exclusion criteria

Papers were excluded from the review if the intervention was directed at the healthcare providers behaviour, or intended for group delivery. Interventions intended for group delivery were considered outside of the scope of this review. The mode of delivery considered for inclusion within this review was selected to best reflect and therefore have increased relevance to the tailored approach to diabetic foot care commonly implemented clinical practice. Book reviews and commentaries were not included.

### Information sources

Searches were conducted both electronically and manually. The search strategy comprised of three steps. First, an initial search was undertaken using the following electronic databases: MEDLINE, CINAHL, AMED, Embase, Cochrane Database of Systematic Reviews, PsycINFO. A second search for unpublished studies included Google Scholar. Finally, a manual search of reference lists was conducted. The reference lists of the selected articles were scanned to identify further studies of potential interest.

Search terms included Diabet* AND foot OR peripheral AND neuropathy, Self-management OR self-management OR self-assessment OR patient centred (centered) OR education OR training OR behaviour* OR motivate*, Prevention AND ulcer* OR Foot AND protection OR adherence OR lifestyle change. The MEDLINE search strategy is provided as Additional File 1.

Three independent reviewers (JP, SA, PH) scanned the title and abstract of the articles identified by the searches to select those matching the inclusion criteria. Full texts of the remaining articles was retrieved. Any disagreement in the final selection of articles of data extraction was resolved through discussion.

### Data items

The key information extracted from each paper was charted using a standardised data extraction tool developed and piloted for this study. The tool was used to capture information to describe the extent, nature and distribution of studies (for example, foot health actions targeted by the intervention, study context, research methods and outcomes, and recipient group characteristics). In addition, data relating to intervention characteristics (eg intervention type, intervention function, mechanism of action and evidence of effectiveness) was independently extracted and charted by the three reviewers. Disagreements were resolved through discussion between the three reviewers (JP, SA, PH) and by further adjudication by a forth reviewer (LC) with specific expertise in the pschcology of behaviour change.

### Collating, summarizing and reporting the results

Studies were grouped to provide a broad view of the extent, nature and distribution of studies included within the review. This information was used to contextualise the data, and identify dominant areas of research activity and subsequently gaps in research activity.

Studies and intervention components specifying a behaviour change related to foot self-management were mapped thematically to classify the behaviour change. In addition, each intervention component was mapped and coded against the COM-B model of behaviour [29]. As anticipated at the outset, many interventions comprised of several sources of behaviour, and each source of behaviour served several intervention functions. In this situation, all sources of behaviour within an intervention were charted, but only the primary intervention function for each source of behaviour was captured. Once charted the data could be synthesised to determine the sources of behaviour and primary intervention functions shared between interventions found to be effective in changing behaviour to improve the foot self-management of people with diabetes.

## Results

### Description of studies

**Fig. 2:**
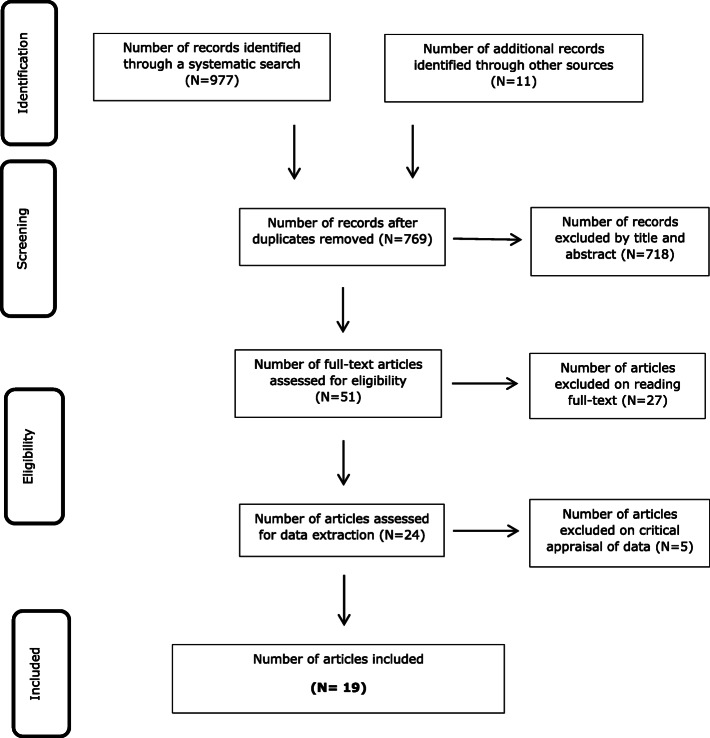
PRISMA [33] flow diagram of search and study selection process

The PRISMA flow diagram details identification and selection of studies (Fig. 2). All citations, abstracts, and papers were independently scanned by three investigators (JP and SA or PH). The initial search produced 988 papers including 219 duplications. Screening by title and abstract of the remaining 769 citations identified 71 potentially relevant articles. Thirty-one articles were retrieved and 41 required further discussion. Of these, 21 papers were excluded and 20 full text articles retrieved. In total 51 potentially relevant full text articles were retrieved. Twenty-seven articles were excluded after reading full text. The primary reasons retrieved studies were excluded from the review were because the studies investigated group interventions. A further five papers were excluded at the data collection stage. A total of 19 papers reporting on 18 studies were included in the review.

### Mapping of results

#### Geographical distribution of studies

**Fig. 3:**
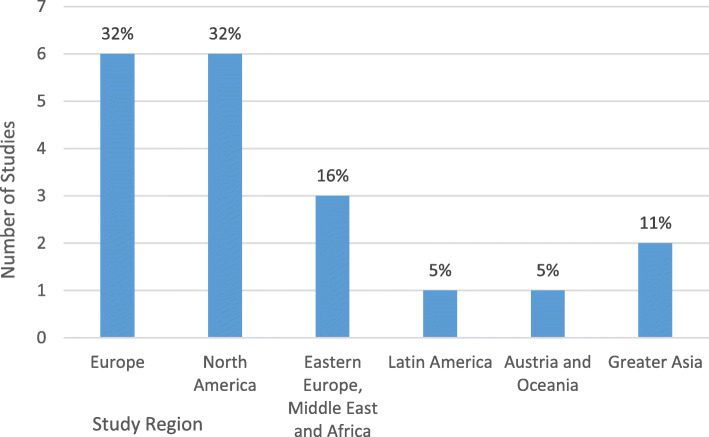
Geographical distribution of studies

The geographical distribution of studies shows the number and percentage of research reports that evaluated interventions, to improve foot self-management behaviour for foot ulcer prevention in people with diabetes, according to the world region in which the intervention were implemented (Fig. 3). The majority of papers described interventions were carried out in North America (32%) [11, 14, 15, 19, 34] or Europe [12, 17, 18, 20, 35, 36] (32%), including a single study from the UK [36]. Iran and Turkey accounted for the 16% of studies in the Eastern Europe Middle East and Africa Region [16, 37]. A Latin America study was implemented in Brazil [13]. China accounted for the two studies implemented in Asia [38, 39].

#### Care setting distribution of studies according to recipient risk of ulceration

**Fig. 4:**
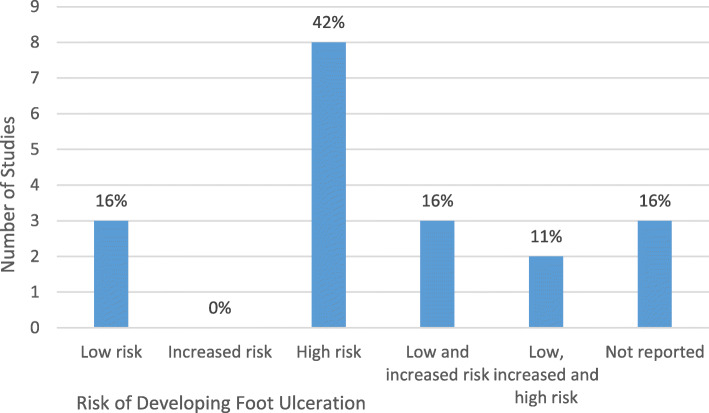
Number and proportion of studies categorised according to participant risk of foot ulceration

Figure 4. shows the number and percentage of studies categorised by study participant diabetic foot ulcer risk status. Three studies (18%) did not report the diabetic foot ulcer risk of the recipients recruited [12, 16, 34]. Emphasis on improving the foot self-management behaviour for foot ulcer prevention in those at high risk of diabetic foot ulceration was evident in 42% of the studies [17, 19, 20, 35, 36, 38–40]. All studies recruiting high-risk participants were conducted within the Hospital setting [17, 19, 20, 38–40] (Table 1). However, the majority of studies (80%), where recipients from the high risk category were excluded, were based within the community [11, 14, 15, 18]. No studies were identified with specific focus on participants at risk of a first foot ulceration. The mean age of study participants categorised as low and increased risk for foot ulceration was 55 years. Study recipients at high risk of ulceration tended to be older (mean 63 years).

**Table 1:** Care setting distribution of studies according to recipient risk of ulceration

	Low	Low and Increased	High	Not reported
Hospital			6	2
Community	2	4	1	2
Home			1	
University Clinic		1		

#### Type of research methods used to evaluate interventions to improve foot self-management in people with diabetes

Most studies (93%) used a quantitative methodology. Of these, twelve of the studies used a randomised control trial design [12, 14, 17, 19, 20, 34, 36–38, 40–42], whilst five were uncontrolled before and after studies [11, 13, 16, 35, 39]. Just one study used mixed methods employing quantitative outcome measures alongside semi-structured interview [15].

#### Sample size

The sample size of the intervention group recruited to the studies varied considerably irrespective of research design with one RCT recruiting 5 participants [17] and another recruiting 267 [18]. The mean number of participants enrolled into the intervention group was 86.

#### Measures of effectiveness

The outcome measures focussed on levels of knowledge, change in behaviour and foot health. The majority of studies developed their own scales to measure levels of knowledge [12, 14, 15, 18, 34, 38, 39], technical competence to carry out a skill or log the regularity with which a desired foot care behaviour was conducted [17, 19, 20, 35, 36, 40, 42]. A small number of studies used standardised ‘off the shelf’ measures of self-efficacy [14, 34, 39]. Half of all studies (*n* = 9) included a measure of foot health [13, 19, 20, 35–37, 40, 42, 43]. Measures of foot health most often concentrated on improved foot function or incidence of foot ulceration [13, 19, 20, 35–38, 40, 42]. One study recorded the number of minor skin lesions as measure of effectiveness [43].

#### Categorising the intervention

The category definitions were taken from the literature [44], to differentiate between interventions that deliver traditional patient education and those that provide self-management education. Traditional patient education was defined as the provision of disease-specific information or skills intended to increase patient compliance through improved technical knowledge and skills (for example, how to cut toe nails safely or avoid walking without shoes). Self-management education in contrast, was defined as the provision of skills necessary to act on problems to improve health outcomes through increased self-efficacy (for example, how to self-monitor feet, understand when a condition is medically serious, how to seek help, and have the confidence to take action). Most foot self-management interventions (56%) delivered traditional foot health education information and skills [12, 13, 17, 34, 35, 37, 38, 41, 42] (Table 2). One study provided self-management education only [19]. In this study, participants were taught how to self-monitor and log skin temperature and when they should reduce activity levels and contact a professional. The remaining studies (38%) used a blend of education strategies to include diabetic foot care education, alongside managing skills (such as, inspecting feet daily and when to seek professional help) [14–16, 20, 38, 39, 43, 45].

**Table 2:** Number and proportion of studies according to intervention category

Categories	All studies		Theory driven interventions	
	N (18)	%	N (3)	%
**Traditional patient education**	10	56	1	6
**Self-management education**	1	6	0	–
**Both**	7	38	2	13

Interventions were categorised as theory driven such as, intervention development underpinned by a conceptual behaviour change model or delivered applying the principles of health coaching. In this context the definition for health coaching was taken from the Better Conversation, Health Coaching website supported by the NHS Innovation Accelerator Programme (https://www.betterconversation.co.uk/). A health coaching approach is defined as a patient-centred process. It entails goal setting determined by the patient, encourages self-discovery, in addition to content education, and incorporates mechanisms for developing accountability in health behaviours. Only three studies (19%) explicitly embraced this patient-centred approach to improving diabetic self-management (Table 2) [17, 34, 43]. One used motivational interviewing to facilitate increased internal motivation within participants, to increase insole adherence [17]. The other two were grounded within the conceptual framework of the Social Cognitive Theory [34, 43]. Both interventions were designed to change foot care behaviour through improved diabetes self-efficacy.

#### Categorising foot health actions (Table 3)

There were many different foot health actions incorporated within the interventions included in this review. Despite the review being based on just 18 research studies, a total of 25 different foot health actions were evaluated. Once a list of foot health actions had been extracted from the literature, to organise the data more meaningfully and to better identify trends or gaps in the evidence, foot health actions were grouped into three broad behaviour orientated headings through a process of theming:: 1. routine self-management, 2. trauma avoidance and foot protection, 3. warning signs and taking action. Those interventions targeted at improving routine self-management (for instance ‘wash and dry feet daily’) were more likely to be delivered as measurable goals or skill-based tasks. In contrast interventions focused on reducing risky behaviour for trauma avoidance or foot protection tended to be framed in more general terms (for instance ‘wear shoes that fit’). Eleven studies (over 60%) were aimed at improving self-screening for early signs of foot problems [11, 13, 14, 16, 18, 19, 34, 36, 38, 39] (Table 3). Several of these failed to recognise that improved self-screening is likely to have limited beneficial impact on health outcomes unless combined with associated self-management foot health activities such as decision making and taking action. For example whilst four studies included self-screening, authors omitted to include the critical decision making element around ‘when and how to seek help’ [13, 34, 38, 39]. Likewise, on finding early signs of foot problems, all but two failed to include any sort of damage limitation activity such as ‘reducing activity levels’ [19, 20] .

**Table 3:** Range and frequency of foot self-management actions and risk behaviours across interventions

		Routine self care								Trauma avoidance													Warning signs and actions			
		Daily technical skills				General technical skills				Daily risk prevention measures								General risk prevention measures					Self screening		Decision making	
Author	Year	moisturise daily	Wash and or dry feet daily	Massage feet daily	Stretching and strengthening exercises	Dry or no moisturiser between toes	Dry feet	cut or file nails regularly	Gentle debridement of callus	Wear cotton socks	wear socks that fit	wear shoes that fit,	Use closed shoes	Examine shoes for cracks	Wear socks when wearing shoes	Inspect inside shoes	wear insoles all of the day time	Avoid using abrasives	never walk barefoot	get corns treated by a podiatrist	Get your feet checked by a professional once a year	keep useful numbers handy	Record foot temp daily	Inspect feet daily	When to reduce activity levels	When to seek professional help
Borges	2008	x				x	x	x			x	x		x				x	x					x		
Cerrahoglu	2016				x																					
Chantelau	1994											x					x									
Lifeeng Fan	2013	x	x	x	x	x	x	x			x	x									x			x		x
Lifeng Fan	2013																									
Hamalainene	1998	x	x		x			x											x	x						
Iunes	2014	x			x	x	x	x		x			x					x	x					x		
Keukenkamp	2018											x					x		x							
Kruger	1992		x					x	x			x												x		x
Lavery	2007																						x	x	x	x
Lavery	2012											x					x									
Ledda	1997	x						x														x		x		x
Liang	2012	x	x																					x		
Lincoln	2008	x	x				x												x					x		x
Ronnemaa	1997	x	x		x			x	x			x			x				x	x						
Skafield	2015											x											x	x	x	x
Li	2018	x	x			x	x	x		x		x				x			x		x			x		
Searle	2019				x																					
Vatankhah	2009	x	x					x					x		x									x		x

Most studies (68%) included interventions addressing foot health actions from two or more of the categories [11–16, 18, 20, 34, 36, 38, 39]. The intervention implemented in eight of these studies comprised of a comprehensive foot self-management improvement package, encompassing foot health actions from all three categories (routine self-management, trauma avoidance and foot protection, warning signs and decision making) [11, 13–16, 34, 36, 39]. The number of foot care actions combined within each of these interventions ranged from five to twelve. Twenty-five percent of studies took a more focused approach to behaviour change intervention design, targeting just one category of foot health activities [17, 19, 35, 37, 40, 42]. The number of activities combined within each of these interventions ranged from one to four. The type of foot health activities associated with this more focused intervention approach were largely aimed at increasing participant adherence to medical devices, specifically therapeutic footwear and insoles for foot protection and skin thermometers for self-monitoring changes in foot temperature [17, 19, 35, 40].

The most frequently targeted and therefore arguably the most important foot health action across all categories was ‘inspect feet daily’. The remaining most frequently targeted foot health actions were all positioned with the routine care category and included moisturise feet daily, wash and dry feet daily, cut or file nails regularly. ‘Wear shoes that fit’ and the inversely phased ‘never walk barefoot’ appeared to be the only foot health activity with consensus between studies from the trauma avoidance and foot protection category. Within this category there were a number of foot health activities with a low rate of occurrence. Most of these were inconsistent foot health activities related to sock choice or fit.

#### Mapping intervention functions and sources of behaviour change

**Fig. 5:**
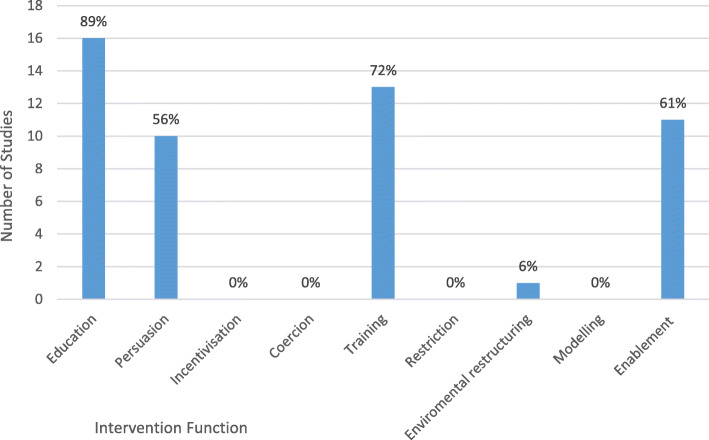
Number and proportion of studies categorised according to intervention function

The COM-B model provides a systematic method of identifying nine possible intervention functions enabling a given intervention or target behaviour (Fig. 1) [28]. Intervention functions were identified and compared across studies to determine which were most commonly employed in footcare self-management interventions [28]. Figure 5. Show the frequency of intervention functions across all interventions for foot protection. Four intervention functions were clearly better suited to this type of behaviour change intervention. Education: increasing knowledge or understanding. Persuasion: Using communication to induce positive or negative feelings or stimulate action. Training: Imparting skills. Enablement: Increase means to increase capability.

The intervention functions form part of the COM-B as a framework for understanding behaviour. Each intervention function can be mapped against one of three essential sources of behaviour (capability, opportunity and motivation). Interventions change behaviour by addressing deficits in one of more of these interacting sources of behaviour (Fig. 1) [28].

**Fig. 6:**
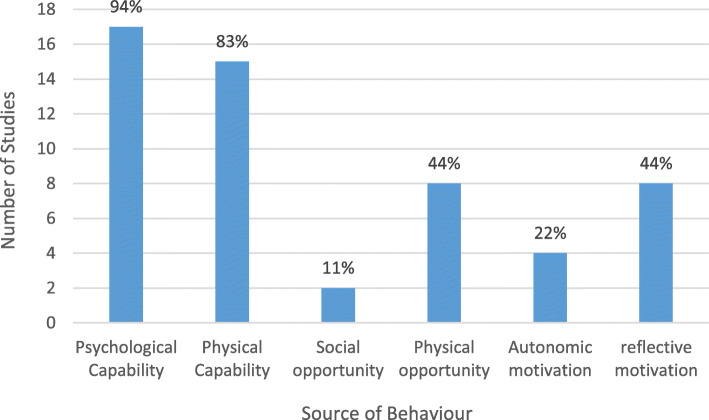
Number and proportion of studies categorised according to sources of behaviour. Detailed legend: The following descriptors are taken from Michie [29]. 1. Physical capability can be achieved through physical skill development with is the focus of training or potentially through enabling interventions such as medication surgery or prostheses. 2. Psychological capability can be achieved through imparting knowledge or understanding training emotional cognitive and / or behavioural sills or through enabling interventions such as medication. 3. Reflective motivation can be achieved through increasing knowledge and understanding eliciting positive (or negative) feelings about behavioural target. 4. Autonomic motivation can be achieved through associative learning that elicit positive (or negative feelings and impulses and counter-impulses relating to the behavioural target, imitative learning, habit formation or direct influences on automatic motivational processes. 5. Physical and social opportunity can be achieved through environmental change [29]

Figure 6 shows that capability (Psychological and Physical) were behavioural deficits most frequently addressed by interventions.

The table shows the frequency of intervention functions mapped against the COM-B model sources of behaviour across all interventions for foot protection (Table 4). Capacity-Psychological/Education and Capacity-Physical/Training were the intervention function and source of behaviour used with most frequency within interventions for foot protection. This was predominantly in combination with enablement to create Opportunity-Physical and/or persuasion to illicit Motivation-reflective. Sources of behaviour infrequently used were Opportunity (Social) and Motivation (Autonomic). Intervention functions never or rarely employed in interventions for foot protection included; incentivisation, coercion, modelling, restriction and environmental restructuring.

**Table 4:** Frequency of intervention functions mapped against sources of behaviour across all interventions for foot protection

Model of behaviour; sources	Education	Persuasion	Incentivisation	Coercion	Training	Restriction	Environmental restructuring	Modelling	Enablement
**C-Ph**					21				
**C-Ps**	19				4				2
**M-Re**	2	13		0					
**M-Au**		0	0	0			1	0	3
**O-Ph**					1	0	1		10
**O-So**						0	0		2

Sub-group behaviour change analysis was conducted on the very small number of interventions reporting a significant result, to determine which intervention functions are most effective in changing foot care behaviour. Interventions that improved participant knowledge all included education and enablement as the intervention function [12, 15, 16, 18, 34, 38]. Interventions that were successful in changing behaviour included education and either enablement training or persuasion [12, 16, 17, 19, 20, 34, 36, 38, 39]. Interventions that resulted in improvements in foot health all contained training and education with the addition of either persuasion or enablement [18, 19].

## Discussion

The aim of this scoping review was first to identify the range of recommended foot self-management actions or risky foot health behaviour modifications for foot ulcer prevention, tested in evaluation studies. This information provides insight into areas of foot self-management with an evidence-base to support its efficacy and identify those areas where to date no research has been conducted. The second part of this review was designed to map the component parts of each foot self-management intervention against the COM-B framework, to define the function of the intervention in terms of its source of behaviour change. In addition, the mapping exercise provides context information regarding geographical location, healthcare setting, healthcare provider of the included study, details of the recipient group, in terms of diabetes disease severity, and the nature of the studies, including research methods adopted and measures of effectiveness.

Most of the included studies took a ‘scattergun’ approach to improving foot care self-management by electing to evaluate multidimensional interventions, specifying up to 12 behaviours [12–16, 18, 20, 34, 36, 38] which makes determining the value of each variable to ulcer prevention impossible. Many interventions failed to target the correct behaviour that needed to change to prevent foot ulceration. Whilst ten studies provided education on self-screening for warning signs for foot problems [11, 13, 14, 16, 18, 34, 36, 38], only two included the necessary decision-making, and action to be taken, as part of the target behaviour [13, 34]. Most emphasis was placed on ulcer detection rather than prevention. Twelve interventions included elements on reducing risky behaviour for trauma avoidance for foot protection (three targeted wearing insoles) [11, 13–16, 18, 34, 36]. However, the majority of behaviours were ill-defined. For example, cutting toe nails frequently and wearing shoes that fit. ‘Inspect feet daily’ received greatest attention from the literature.

Interventions were often aimed at those at high risk of foot ulceration (41%) [19, 20, 35, 36, 38, 40] or included people from the low risk category (36%) [11, 14, 15, 18]. The remaining studies did not specify participant diabetic foot ulcer risk status within their report. No studies targeted people with diabetes at risk of developing a first foot ulceration. Outcome measures were not comparable between studies. However, seven studies (41%) used participant knowledge levels as a measure of intervention effectiveness [11, 12, 14, 16, 18, 34, 38]. Incidence of ulceration, amputation, incidence of minor foot problems, and prevalence of foot conditions, foot temperature and visits to the podiatrist were outcome measures all used to assess foot health [11, 18, 20, 35, 36, 38, 40]. No studies included an economic component. To achieve clinical impact, future definitive randomised control trials should include incidence of ulceration as their primary outcome. Secondary short-term outcome measures such as knowledge levels and behaviour change are useful to give insight into the mechanism of action for intervention development, and as an indicator of intervention potential prior to proceeding an intervention to a full trial. Authors should be encouraged to develop innovative approaches for the assessment of self-management interventions which draw upon the patient experience and expertise to ensure that interventions best meet the needs of people living with diabetes. In particular outcome measures which capture affordability, practicality of service delivery within clinical context, acceptability to patients to optimise attendance and adherence, and equity for patients with disparities in health and from different social sectors.

The majority of foot care self-management interventions (88%) are delivered using didactic, professional-centred health education [11–16, 18, 34, 36]. This tends to cover skill-based tasks such as self-screening, routine foot care and general hygiene. Whilst this approach can be successful in improving knowledge and foot self-management skills, participant decision-making abilities around when to seek professional help or reduce activity levels was not seen to improve. Possibly because behaviour change determinants (capability, opportunity and motivation) were not being addressed in the intervention design.

Most papers lacked detail in their description of intervention content, method of development and intervention delivery. Only three studies evaluated foot care self-management interventions developed using a behavioural change framework [17, 34, 46]. Only three studies embraced a patient centred approach to improving diabetic foot self-management and its delivery for foot ulcer prevention [17, 34, 43]. Research favouring a patient centred approach or underpinned by a theoretical behaviour change framework used ‘change in participant behaviour’ as the preferred outcome measure. Intervention delivery was inconsistent; sessions ranged from one 15-min contact, to 13 contacts over a one-year period.

The evidence was charted against the COM-B model to reveal a core set of components from the behaviour change model best suited for the development of interventions to promote foot self-management. This core set of components are Psychological Capability (necessary knowledge), Physical Opportunity (necessary time and resources) and Reflective Motivation (intent and beliefs about consequences). These core components were inked to four intervention functions. The two key combinations of the four intervention functions identified as most likely to bring about a change in foot self-management behaviour are 1. Education (increasing knowledge of their diabetes and foot self-management) and skills training (imparting physical foot self-management skills and techniques) in combination with persuasion (using communication to induce positive feelings about foot management or negative feelings about foot ulceration as a consequences of undesirable behaviour), and 2. Education and skills training in combination with enablement (increasing capability or opportunity by providing cues, prompts or equipment).

### Gaps and recommendations for future research

This review noted a number of potential areas for future research to advance the development of diabetic foot self-management management interventions. Intervention development could be accelerated through the employment of robust, systematic approaches to intervention design. This would place emphasis on defining the specific foot care self-management behaviour for change, the impact of that change on ulceration risk and the extent to which the behaviour can be measured. This development work will require innovative approaches to design, implementation and assessment interventions, which draw upon the patient’s experience and expertise.

To make a decision on best practice, randomised controlled trials implemented over an extended follow up period are required to determine which combination of behaviour change techniques, underpinned by a theoretical behaviour change framework, are effective, and cost-effective, in supporting foot self-management behaviour for preventing foot ulceration. Clinical trials should use sound methodologies to examine the best way to put into practice (context, delivery format) and measure (outcome measures) foot care self-management interventions. The development and application of standard outcome measures to determine the effectiveness of an intervention for foot ulcer prevention would enable better evaluation and comparison.

### Limitations

Several limitations for this scoping review should be acknowledged. Publications not in the English language were excluded, potentially reducing the available data. Additionally, although the main search bases, reference lists, biographies and google scholar were recruited, some of the grey literature may have been missed. Therefore, this review may not have exhausted all sources of data. It is also recognised that the research team lacked expertise in behaviour change, to reduce the risk of bias, a behavioural psychologist provided expert oversight, and mentorship for this area of the work.

## Conclusion

The self-management actions or risky behaviour modifications targeted by most studies evaluating behaviour change interventions for improving foot self-management for diabetic foot ulcer prevention appear to focus on an ad-hoc combination of self-screening and skill based foot care tasks. To increase impact on foot health outcomes intervention designers should shift emphasis toward empowering and building patient confidence to enable sound decision making around taking preventative action or seeking professional help when warning signs or ulceration are discovered.

With reference to the COM-B model, to understand behaviour, to have the greatest chance of success interventions to support diabetic foot self-management should be underpinned by behaviour change theory and aim to address all determinants of behaviour; capability, opportunity and motivation. Charting the evidence revealed a core set of intervention functions common to interventions effective in improving foot self-management behaviour (education and training and at least one other: persuasion or enablement).

A number of research gaps were identified through conducting this review. Randomised controlled trials with an extended follow up are particularly required, targeting those yet to develop first diabetic foot ulceration. Development of a core set of minimum outcome measures for clinical trials for diabetic foot ulcer prevention are required to reduce the social and economic impact of foot ulceration globally.

## Supplementary Information

**Additional file 1.**

**Additional file 2.**

**Additional file 3.**

## Data Availability

The datasets used and/or analysed during the current study are available from the corresponding author on reasonable request.
