# Supplementary material for: Behaviour change approaches for individuals with diabetes to improve foot self-management: a scoping review
Source: J Foot Ankle Res. 2021 Jan 6;14:1. doi: 10.1186/s13047-020-00440-w (PMC7788877; doi:10.1186/s13047-020-00440-w)
Supplement: Supplementary file 1 — Additional file 1. [file 13047_2020_440_MOESM1_ESM.docx]

Additional file 1.

MEDLINE Search Strategy using OVID

1. Diabetic foot/

2. Diabetic ulcer*. ti,ab

3. Foot ulcer*.ti,ab

4. Diabetic foot health.ti,ab

5. 1 or 2 or 3

6. exp Primary prevention/

7. exp Secondary prevention/

8. 6 or 7

9. exp Health promotion/

10. self-examination/

11. self-management/

12. self-care/

13. motivational interviewing/

14. motivation/

15. patient compliance/

16. patient non-compliance/

17. patient education/

18. Risk reduction behaviour/

19. adherence. ti,ab

20. behaviour adj change.ti,ab

21. patient adj (centered or centred).ti,ab

22. 9 or 10 or 11 or 12 or 13 or 14 or15 or 16 or 17 or 18 or 19 or 20 or 21

23. 5 and 8 and 23
